# Genetic Manipulation of *Schistosoma haematobium*, the Neglected Schistosome

**DOI:** 10.1371/journal.pntd.0001348

**Published:** 2011-10-11

**Authors:** Gabriel Rinaldi, Tunika I. Okatcha, Anastas Popratiloff, Mary A. Ayuk, Sutas Suttiprapa, Victoria H. Mann, Yung-san Liang, Fred A. Lewis, Alex Loukas, Paul J. Brindley

**Affiliations:** 1 Department of Microbiology, Immunology and Tropical Medicine, School of Medicine and Health Sciences, The George Washington University Medical Center, Washington, DC, United States of America; 2 Departamento de Genética, Facultad de Medicina, Universidad de la República, (UDELAR), Montevideo, Uruguay; 3 Department of Tropical Medicine, School of Public Health and Tropical Medicine, Tulane University, New Orleans, Louisiana, United States of America; 4 Center for Microscopy and Image Analysis, The George Washington University, Washington, DC, United States of America; 5 Biomedical Research Institute, Rockville, Maryland, United States of America; 6 Queensland Tropical Health Alliance, James Cook University, Cairns, Queensland, Australia; University of Queensland, Australia

## Abstract

**Background:**

Minimal information on the genome and proteome of *Schistosoma haematobium* is available, in marked contrast to the situation with the other major species of human schistosomes for which draft genome sequences have been reported. Accordingly, little is known about functional genomics in *S. haematobium*, including the utility or not of RNA interference techniques that, if available, promise to guide development of new interventions for schistosomiasis haematobia.

**Methods/Findings:**

Here we isolated and cultured developmental stages of *S. haematobium*, derived from experimentally infected hamsters. Targeting different developmental stages, we investigated the utility of soaking and/or square wave electroporation in order to transfect *S. haematobium* with nucleic acid reporters including Cy3-labeled small RNAs, messenger RNA encoding firefly luciferase, and short interfering RNAs (siRNAs). Three hours after incubation of *S. haematobium* eggs in 50 ng/µl Cy3-labeled siRNA, fluorescent foci were evident indicating that labeled siRNA had penetrated into miracidia developing within the egg shell. Firefly luciferase activity was detected three hours after square wave electroporation of the schistosome eggs and adult worms in 150 ng/µl of mRNA. RNA interference knockdown (silencing) of reporter luciferase activity was seen following the introduction of dsRNA specific for luciferase mRNA in eggs, schistosomules and mixed sex adults. Moreover, introduction of an endogenous gene-specific siRNA into adult schistosomes silenced transcription of tetraspanin 2 (*Sh-tsp-2*), the apparent orthologue of the *Schistosoma mansoni* gene *Sm-tsp-2* which encodes the surface localized structural and signaling protein Sm-TSP-2. Together, knockdown of reporter luciferase and *Sh-tsp-2* indicated the presence of an intact RNAi pathway in *S. haematobium*. Also, we employed laser scanning confocal microscopy to view the adult stages of *S. haematobium*.

**Conclusions:**

These findings and approaches should facilitate analysis of gene function in *S. haematobium*, which in turn could facilitate the characterization of prospective intervention targets for this neglected tropical disease pathogen.

## Author Summary

More people are infected with *Schistosoma haematobium* than other major human schistosomes yet it has been less studied because of difficulty in maintaining the life cycle in the laboratory. *S. haematobium* might be considered the ‘neglected schistosome’ since minimal information on the genome and proteome of *S. haematobium* is available, in marked contrast to the other major schistosomes. In this report we describe tools and protocols to investigate the genome and genetics of this neglected schistosome. We cultured developmental stages of *S. haematobium*, and investigated the utility of introducing gene probes into the parasites to silence two model genes. One of these, firefly luciferase, was a reporter gene whereas the second was a schistosome gene encoding a surface protein, termed *Sh-tsp-2*. We observed that both genes could be silenced – a phenomenon known as experimental RNA interference (RNAi). These findings indicated that the genome of *S. haematobium* will be amenable to genetic manipulation investigations designed to determine the function and importance of genes of this schistosome and to investigate for novel anti-parasite treatments.

## Introduction

More people are infected with *Schistosoma haematobium* than with the other schistosomes combined. Of >110 million cases of *S. haematobium* infection in sub-Saharan Africa, 70 million are associated with hematuria, 18 million with major bladder wall pathology, and 10 million with hydronephrosis leading to severe kidney disease [1], [2], [3]. In many patients, chronic inflammation in response to *S. haematobium* ova leads to squamous cell carcinoma of the bladder [4], [5]. *S. haematobium* is classified as a Group 1 carcinogen by the World Health Organization's International Agency for Research on Cancer [6], [7] although the cellular and/or molecular mechanisms linking *S. haematobium* infection with cancer formation have yet to be defined [8]. One quarter to three quarters of women infected with *S. haematobium* suffer from female genital schistosomiasis (FGS) of the lower genital tract [1]. FGS results from deposition of the schistosome eggs in the uterus, cervix, vagina and/or vulva, with ensuing host inflammatory responses comprised of granulomas, fibrosis, and pathological localized blood vessel formation [9]. FGS increases susceptibility to HIV/AIDS [10], [11], [12], and decreases female fertility [13].

Given the enormous numbers of people infected with S. *haematobium*, and the pathogenesis of *S. haematobium* infection, including its association with bladder cancer and HIV/AIDS, there is a pressing need for new approaches to control including the development of a vaccine to prevent infection with *S. haematobium*. With regard to fundamental aspects of the host-parasite relationship, research on *S. haematobium* is in its infancy compared to *S. mansoni* and *S. japonicum* [14]. There have been massive recent advances in genomic, transcriptomic, and proteomic datasets for both *S. japonicum* and *S. mansoni* [15], [16], [17]. There now is an urgent need to establish similar datasets for *S. haematobium*, and in addition to establish tools and approaches to determine the function and importance of these schistosome genes - including *S. haematobium*-specific genes [14]. Here we cultured several developmental stages of *S. haematobium* and applied several functional genomics approaches to this species. We report that this schistosome, like *S. mansoni* and *S. japonicum*, is amenable to transformation with nucleic acid probes. Notably, the findings indicated the presence of an intact, active RNA interference pathway in *S. haematobium*, the neglected schistosome.

## Materials and Methods

### *Schistosoma haematobium*

Eggs of an Egyptian strain of *S. haematobium* were isolated from either small intestines, that had been thoroughly rinsed in 1× PBS to remove the gut contents, or livers of experimentally infected Syrian golden hamsters [18] following a protocol optimized for isolating eggs of *S. mansoni* from livers of mice [19]. In brief, three to five livers or two to three washed small intestines were chopped finely with a scalpel blade, and then blended to a smooth consistency in 50 ml of phosphate-buffered saline, pH 7.4 (PBS), 5 ml of 0.5% clostridial collagenase (Sigma) and 500 µl of polymyxin B (Sigma). Digests were incubated with gentle shaking at 37°C overnight, after which the contents were subjected to centrifugation at 400×*g* for 5 min. The supernatant was removed and the pellet resuspended in 50 ml PBS. This wash procedure was repeated twice more, with the exception that after the final centrifugation the pellet was resuspended into 25 ml of PBS. The resuspended mixture from liver was passed sequentially through 250 and 150 µm sieves. No passes through sieves were performed with the gut mixture. The liver mixture filtrate or the gut mixture were centrifuged at 400×*g* for 5 min, the supernatant discarded and the pellet resuspended in 3 ml of PBS. This was applied to a column of Percoll, prepared by mixing 8 ml of Percoll (GE Healthcare Bio-Science AB) with 32 ml of 0.25 M sucrose in a 50 ml tube. The tube was centrifuged at 800×*g* for 10 min. Liver or intestinal cells and debris that remained on the top of the Percoll were removed with a Pasteur pipette. The schistosome eggs, which pelleted tightly at the bottom of the tube, were washed three times with PBS and any residual host cells were removed by discarding the supernatant. Further purification of eggs was achieved by resuspension in 0.5 ml of PBS and application on to a second Percoll column, prepared by mixing 2.5 ml of Percoll with 7.5 ml of 0.25 M sucrose in a 15 ml polypropylene tube. The eggs were pelleted and then washed as before. Some eggs were snap frozen and stored at −80°C until use for extraction of total RNA. For other aliquots, the eggs were resuspended in 6 ml of complete culture medium - Dulbecco's modified Eagle's medium (DMEM) with 10% fetal bovine serum (FBS) and 100 U of penicillin and streptomycin (Invitrogen, Carlsbad, CA), split into 2 ml aliquots in a six-well plate and cultured at 37°C under 5% CO_2_.

*S. haematobium* schistosomula were obtained by mechanical transformation of cercariae released from infected *Bulinus truncatus truncatus* snails and cultured at 37°C in modified Basch's medium under 5% CO_2_ in air as described for *S. mansoni* schistosomula [20]. Mixed sex adults of *S. haematobium* were obtained by portal perfusion of infected hamsters followed by mesenteric vessel dissection and manual removal of adult worms using forceps under a magnification glass [18]. The adults were rinsed several times in PBS and cultured in complete culture medium.

### Exposure of *S. haematobium* eggs to Cy3-siRNA

*S. haematobium* eggs were either electroporated and soaked in non-coding Cy3-labeled siRNAs (Silencer Cy3-Labeled Negative Control siRNA, Ambion, Austin, TX) at 50 ng/µl with conditions as described [21]. Briefly, eggs were washed in DMEM supplemented with 200 U/ml penicillin G sulfate, 200 mg/ml streptomycin sulfate, 500 ng/ml amphotericin B, 10 mM HEPES (wash medium) and transfected in 100 µl of the same medium in 4 mm gap cuvettes with an ElectroSquarePorator ECM830 (BTX, San Diego, CA) using a single square wave pulse of 125 volts of 20 milliseconds duration. After electroporation, eggs were washed in PBS three times to remove the unincorporated Cy3-labeled siRNA. Subsequently, eggs were transferred into complete DMEM at 37°C for three hours. Other eggs were soaked for three hours in Cy3-siRNA, then washed in PBS three times in order to remove the unincorporated Cy3-labeled siRNAs. The Cy3-siRNA exposed eggs, with or without electroporation, were examined under bright and fluorescent light (below) using a Zeiss Axio Observer A.1 inverted microscope fitted with a digital camera (AxioCam ICc3, Zeiss). Manipulation of digital images was undertaken with the AxioVision release 4.6.3 software (Zeiss). These manipulations were limited to insertion of scale bars, adjustments of brightness and contrast, cropping and the like; image enhancement algorithms were applied in linear fashion across the entire image and not to selected aspects.

### Synthesis of mRNA, dsRNA, and siRNAs

To synthesize firefly luciferase mRNAs (mLuc), *in vitro* transcriptions of capped RNAs from PCR DNA templates were accomplished using the mMachine T7 Ultra kit (Ambion) as described [22], [23]. Subsequently, RNAs were precipitated with ammonium acetate, dissolved in nuclease-free water and quantified by spectrophotometry (NanoDrop Technologies, Wilmington, DE). The dsRNAs were generated by *in vitro* transcription using, as templates, PCR products amplified with gene specific primers tailed with the T7 promoter sequence. A luciferase dsRNA (dsLuc) template encoding the full length 1,672 kb was amplified from the pGL3-basic plasmid (Promega, Madison, WI), (F: 5′TAATACGACTCACTATAGGGTGCGCCCGCGAACGACATTTA-3′; R: 5′- TAATACGACTCACTATAGGGGCAACCGCTTCCCCGACTTCCTTA-3′). The siRNAs were designed with the assistance of the BLOCK-iT™ RNAi Designer Tool, https://rnaidesigner.invitrogen.com/rnaiexpress/index.jsp. Block-iT™ siRNA of 19 nt in length named si*Sh*TSP 2 (5′-GGA AUC CUG UUU CAA AGA U-3′), specific for residues 159–177 of the extracellular loop 2 of *S. haematobium* tetraspanin 2 (*Sh-tsp-2*) and an irrelevant siRNA (control) termed siScrambled, 5′-GGA GUC CCU UUA AAU AGA U-3′, the sequence of which included the same residues of si*Sh-tsp-2* but in which the order of the residues had been randomly mixed, were purchased from Invitrogen.

### Transfection of developmental stages of *S. haematobium* with mRNA and/or dsRNA

*S. haematobium* eggs were maintained for one day after isolation from hamsters, then subjected to electroporation in the presence of mLuc at 150 ng/µl [21]. Briefly, ∼2,000 eggs were subjected to the square wave electroporation in 4 mm gap pathway cuvettes (BTX) in 100 µl wash medium, as above. A group electroporated in the absence of mLuc was included as a mock-treated control. Thereafter the eggs were kept in culture for 3 or 20 hours, harvested and stored at −80°C. For RNAi approaches, one group of eggs was incubated with 30 µg of dsLuc, and other two groups were incubated without dsLuc. After 10 min at 23°C, 15 µg of mLuc was added to eggs in wash medium, except to a mock control group, i.e. a group of eggs not treated with exogenous nucleic acids. The eggs were subjected to square wave electroporation (above), transferred to pre-warmed culture medium and harvested three hours later.

Schistosomula of *S. haematobium* were removed from culture three hours after cercarial transformation, washed and resuspended in 100 µl of wash medium containing 30 µg of dsLuc. Two other groups of schistosomules were incubated in the absence of dsLuc, in 4 mm gap cuvettes. After 10 min incubation at 23°C, 15 µg of mLuc was added to the wash medium in each group, except to a mock control group. Thereafter the schistosomules were subjected to square wave electroporation, 125 V, 20 ms, transferred to prewarmed Basch's medium and harvested three hours later.

We have recently determined that dicing adult schistosomes into several fragments results in more reporter gene activity than in similar numbers of intact worms [24]. Accordingly, ∼50 mixed sex adults of *S. haematobium* were removed from culture 24 hours after perfusion from hamsters, washed, diced into three or four fragments using a sterile blade. Intact or fragmented *S. haematobium* worms were placed into 4 mm gap pathway cuvettes in the presence of 15 µg of mLuc resuspended in 100 µl of wash medium and subjected to square wave electroporation, 125 V, 20 ms, one pulse. After electroporation, the worms and fragments were transferred into pre-warmed complete culture medium, incubated at 37°C under 5% CO_2_ in air, and harvested 3 hours later.

For RNAi approaches targeting the luciferase reporter gene, the worms were diced into three or four fragments using a sterile blade, washed three times in wash medium and transferred to 4 mm gap cuvettes containing 100 µl wash medium. One group of diced adult worms was incubated with 30 µg of dsLuc, and the other two were incubated in the absence of dsLuc. Following incubation at 23°C for 10 min, 15 µg of mLuc was added to each group, except to the mock control group after which the parasites were subjected to a single pulse of square wave electroporation, 125 V, 20 ms. Subsequently, the diced worms were transferred to complete medium and maintained in culture; the worm fragments remained active (displaying movements) during the study.

For RNAi targeting an endogenous *S. haematobium* gene, intact adult worms were electroporated in the presence of 10 µg of si*Sh-tsp-2* or 10 µg of siScrambled in 100 µl (16.5 µM) of wash medium. We targeted intact worms for this experiment, dealing with silencing of an endogenous gene, with the aim of determining whether a gross phenotype might accompany gene knockdown. After electroporation, worms were transferred to complete medium for 48 h, then stored at −80°C.

### Luciferase activity

Developmental stages of *S. haematobium* were harvested three hours after electroporation unless otherwise indicated, washed three times with wash medium and stored as wet pellets at −80°C. Luciferase activity in extracts of parasites was determined using Promega's luciferase assay reagent system and a tube luminometer (Sirius, Berthold, Pforzheim, Germany) [22]. In brief, pellets of parasites were subjected to sonication (3×5 s bursts for schistosomula and adults and 5×5 s bursts for eggs, output cycle 4, Misonix Sonicator 3000, Newtown, CT) in 300 µl 1× CCLR lysis buffer (Promega). The sonicate was clarified by centrifugation at 20,800 *g*, 15 min, 4°C and the supernatant, containing the soluble fraction, analyzed for luciferase activity. Aliquots of 100 µl of soluble fraction were injected into 100 µl luciferin at 23°C, mixed, and relative light units (RLUs) determined 10 s later by the luminometer. Replicate samples were measured, with results presented as the average of the readings per mg of protein. The protein concentration in the soluble fraction of the schistosome extract was determined using the bicinchoninic acid assay (Pierce, Rockford, IL). Recombinant luciferase (Promega) was included as a positive control.

### Gene expression analysis of *Sh-tsp-2*

Expression of *Sh-tsp-2* mRNA was analyzed in adults of *S. haematobium* harvested 48 hours after RNAi treatment. Total RNA was extracted from the worms using the RNAqueus®-4PCR Kit (Ambion). Any residual DNA remaining in the RNA was removed by DNase digestion using TurboDNase (Ambion) and cDNA was synthesized from 100 ng of total RNA using the iScript cDNA Synthesis Kit (Bio-Rad, Hercules, CA). Primers and TaqMan probes were designed with the assistance of Beacon Designer (Premier Biosoft International, Palo Alto, CA) to obtain probes targeting *Sh-tsp-2* and *S. haematobium* tropomyosin (*Sh*Trop) (GenBank L76202.1) genes, as follows: for *Sh*TSP 2, forward primer: 5′-GAT GCA TTA AGA GAA TTC GTA A- 3′; reverse primer: 5′-TGG TGG AGT GAC ATA ATC-3′; probe: 5′-/56-FAM/TGA AGA ATC AGC ACC ACA GCA TTG/3IABlk_FQ/-3′; for *Sh*Trop, forward primer: 5′-ATC CGA GAT TTA ACA GAA C-3′; reverse primer: 5′-CGC TAA GAG CTT TGT ATC-3′; probe: 5′-/56-FAM/TTC TCA GCC AGT AAG TCA TCT TCC AA/3IABlk_FQ/-3′.Quantitative PCRs were performed in triplicate, using 96-well plates (Bio-Rad), with an initial denaturation step at 95°C for 3 minutes followed by 40 cycles of 30 sec at 95°C and 30 sec at 50°C, using a thermal cycler (iCycler, Bio-Rad) and a Bio-Rad iQ5 detector to scan the plates in real time. Reactions were carried out in 20 µl volumes with primer-probe sets (*Sh-tsp-2*, *Sh*Trop) and Perfecta qPCR FastMix, UNG (Quanta Bioscience, Gaithersburg, MD). The relative quantification assay 2^−ΔΔCt^ method [25] was employed, using *Sh*Trop as the reference gene. Results were plotted as *Sh-tsp-2* gene expression level relative to the reference gene considering 1 = *Sh-tsp-2* relative expression level measured in the irrelevant control group.

### Laser scanning confocal microscopical imaging

Adult flukes were fixed in 4% paraformaldehyde overnight, rinsed with PBS, then incubated in propidium iodine (PI) diluted 1∶1000 for one day. The PI-stained worms were placed on polylysine coated 50 mm Petri dishes, covered with PBS, and examined using a Carl Zeiss LSM 710 confocal system. This system includes a Zeiss Axio Examiner Z1 upright microscope equipped with a 20×/1.0 water dipping objective lens, deployment of which seemed prudent for imaging entire schistosomes since this objective does not require a coverslip (which markedly diminishes spherical aberrations). Confocal images were captured using a Qasar 32-channel spectral detector. Briefly, the worms were simultaneously scanned with 488 and 561 nm laser lines (multiline argon and diode laser, respectively), while the backward light was registered in 1024×1024 lambda-stack images taken simultaneously at a spectral resolution of 9.6 nm. Thus, for each single optical section, 32 images were recorded covering the visible spectrum from 423–721 nm, allowing the analysis of each of the (1) reflected light, (2) autofluorescence, and (3) characteristic emission at 617 nm from PI. To detect the reflected laser light, we utilized a T80/R20 beamsplitter, which only partially attenuates the laser lines in the backward direction. Confocal stacks for three-dimensional (3D) rendering were taken at z-scaling of 1.7 µm, which matched the pinhole opening (34 µm). Pixel resolution was 0.59 µm. After completion of the online acquisition, a linear spectral unmixing protocol was applied to the lambda-stacks to generate two three-channel confocal stacks. To generate reliable spectral unmixed channels, various sites from the worm were tested and representative for the 488-line reflection, autofluorescence and PI were selected and used as reference for unmixing. Thus, the resulting images, encoded in three channels reflected light, autofluorescence and PI signals from the nuclei. Unmixed, confocal stacks were imported to Volocity (v.5.5, Perkin Elmer/Improvision) for further three-dimensional rendering and analysis.

### Ethics statement

Male LVG hamsters were purchased from Charles River (Wilmington, MA) and maintained in the Biomedical Research Institute's (BRI) animal facility, which is accredited by the American Association for Accreditation of Laboratory Animal Care (AAALAC; #000779), is a USDA registered animal facility (51-R-0050), and has an Animal Welfare Assurance on file with the National Institutes of Health, Office of Laboratory Animal Welfare (OLAW), A3080-01. Maintenance of the hamsters, exposure to *S. haematobium* cercariae, and subsequent harvesting of tissues were approved by the BRI Institutional Animal Care and Use Committee (protocol approval number 09-03). All procedures employed were consistent with the Guide for the Care and Use of Laboratory Animals.

## Results

### Culture of developmental stages of *Schistosoma haematobium*

**Figure 1:**
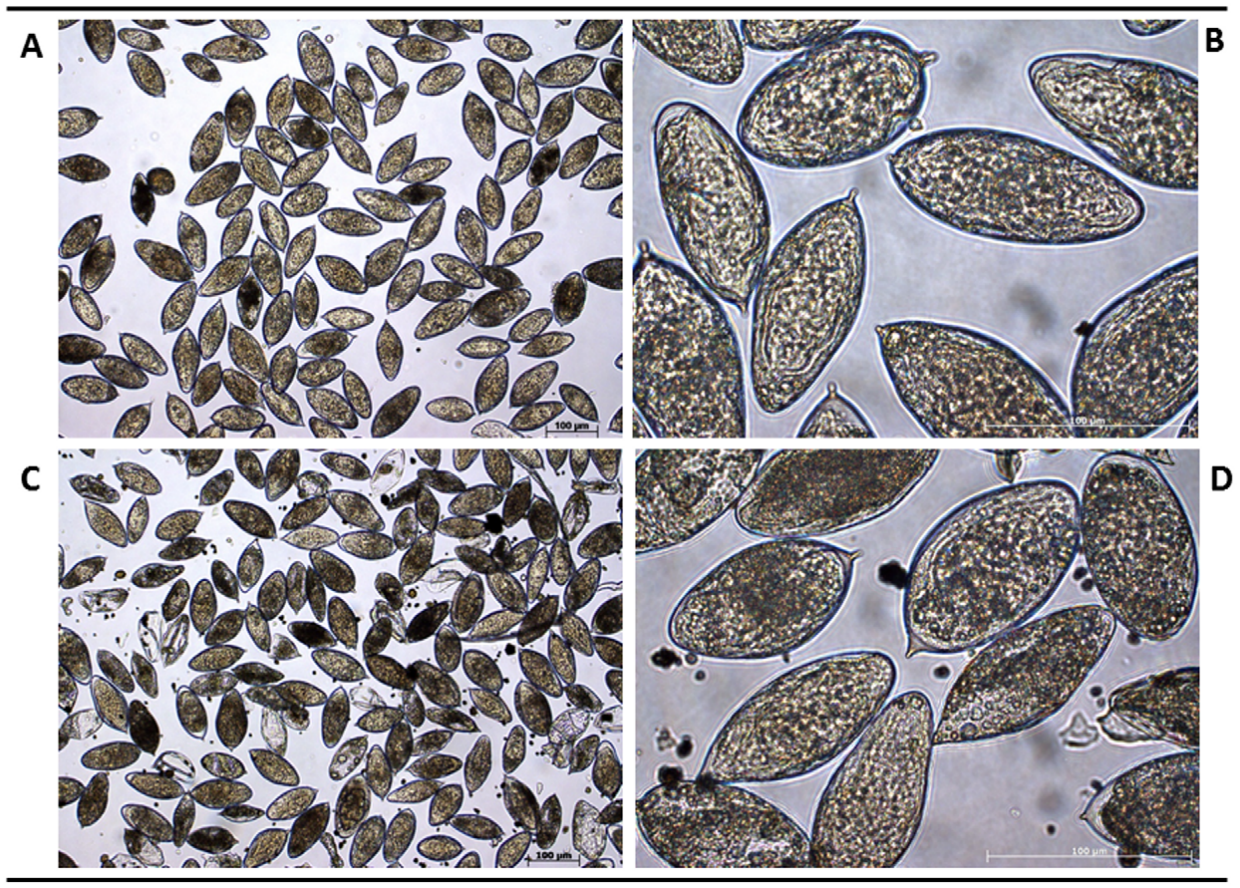
Eggs of *Schistosoma haematobium*. These schistosome eggs were obtained from experimentally infected hamsters, and thereafter were maintained in culture. Eggs were recovered from small intestines (panels A and B) or liver (panels C and D). Scale bars, 100 µm.

**Figure 2:**
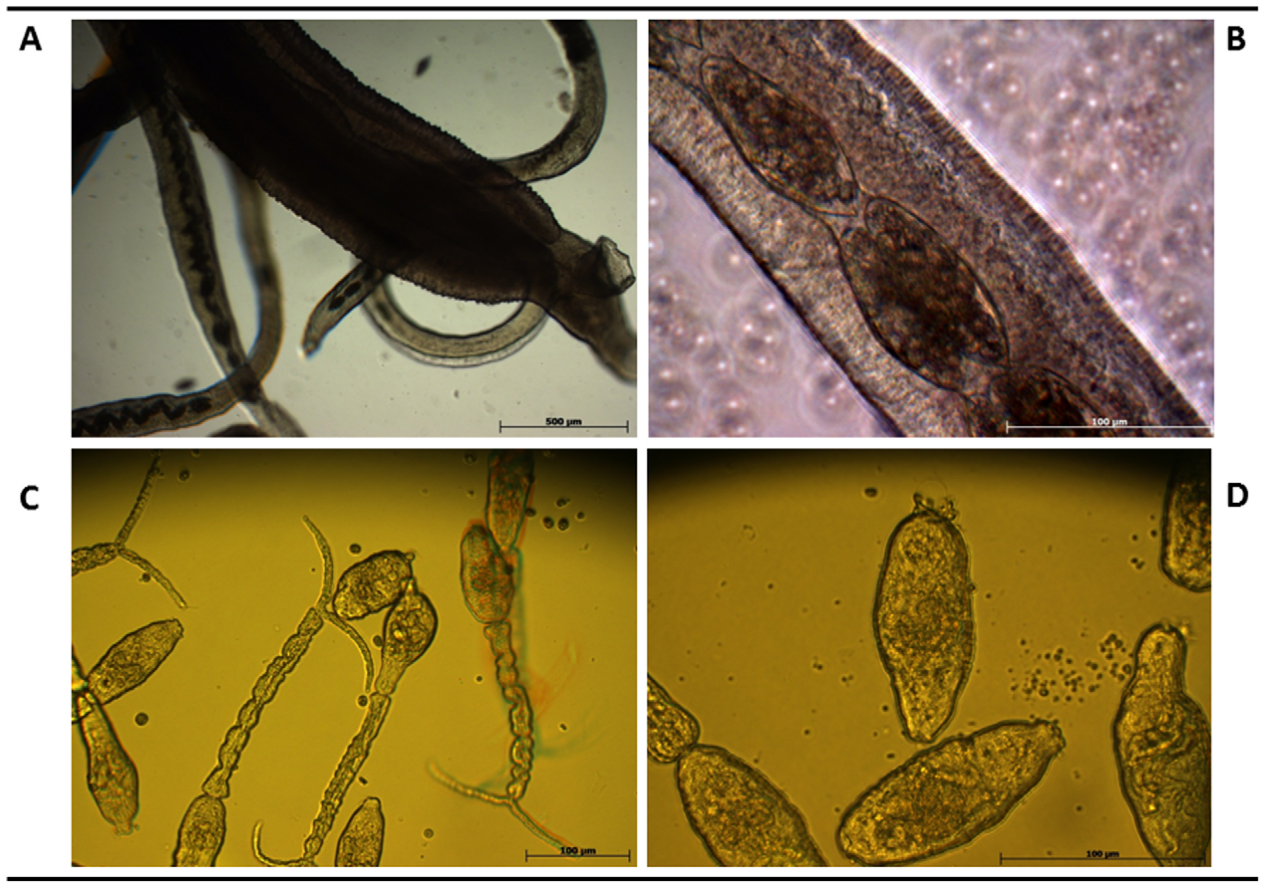
Adults and micrograph showing the characteristic longitudinal disposition of the eggs along the body of the schistosomules of *Schistosoma haematobium*. Panel A: micrograph illustrating a population of mixed sex adults obtained by portal perfusion from infected hamsters and maintained in culture. Panel B: high magnification micrograph showing the characteristic longitudinal disposition of the eggs along the body of the female. Panel C: images of cercariae released from infected snails. Panel D: images of representative schistosomules in culture 3 hours after cercarial transformation. Scale bars, 500 µm (A) and 100 µm (B, C and D).

Given the scarcity of reports on *in vitro* culture techniques focused on *S. haematobium* we adapted protocols from studies with *S. mansoni* [20], to maintain some developmental stages in culture. Thus, eggs isolated either from small intestines or livers of hamsters, schistosomula mechanically transformed from cercariae released by experimentally infected *B. t. truncatus* snails, and mixed sex adults from portal perfusion and mesenteric vessel dissection of hamsters were cultured in the indicated medium at 37°C, 5% CO_2_. No differences in gross appearance were evident between the eggs isolated from intestines (Figure 1A and B) or liver (Figure 1C and D). The eggs were cultured in complete medium (Dulbecco's modified Eagle's medium (DMEM) with 10% fetal bovine serum (FBS) and 100 U of penicillin and streptomycin (Invitrogen, Carlsbad, CA), for up to seven days (not shown). Mixed sex adults were cultured in complete medium (above) for up to five days (Figure 2A). Notably, at higher magnification, longitudinal orientation of the eggs within the uterus of the female schistosome was apparent (Figure 2B) which is in marked contrast to the transverse disposition in utero of *S. mansoni* eggs, e.g. [26], [27]. Schistosomula of *S. haematobium*, obtained by cercarial transformation as described above, were cultured in modified Basch's medium (Figure 2C and 2D).

### Cy3-siRNA is effectively incorporated into eggs of *S. haematobium*

**Figure 3:**
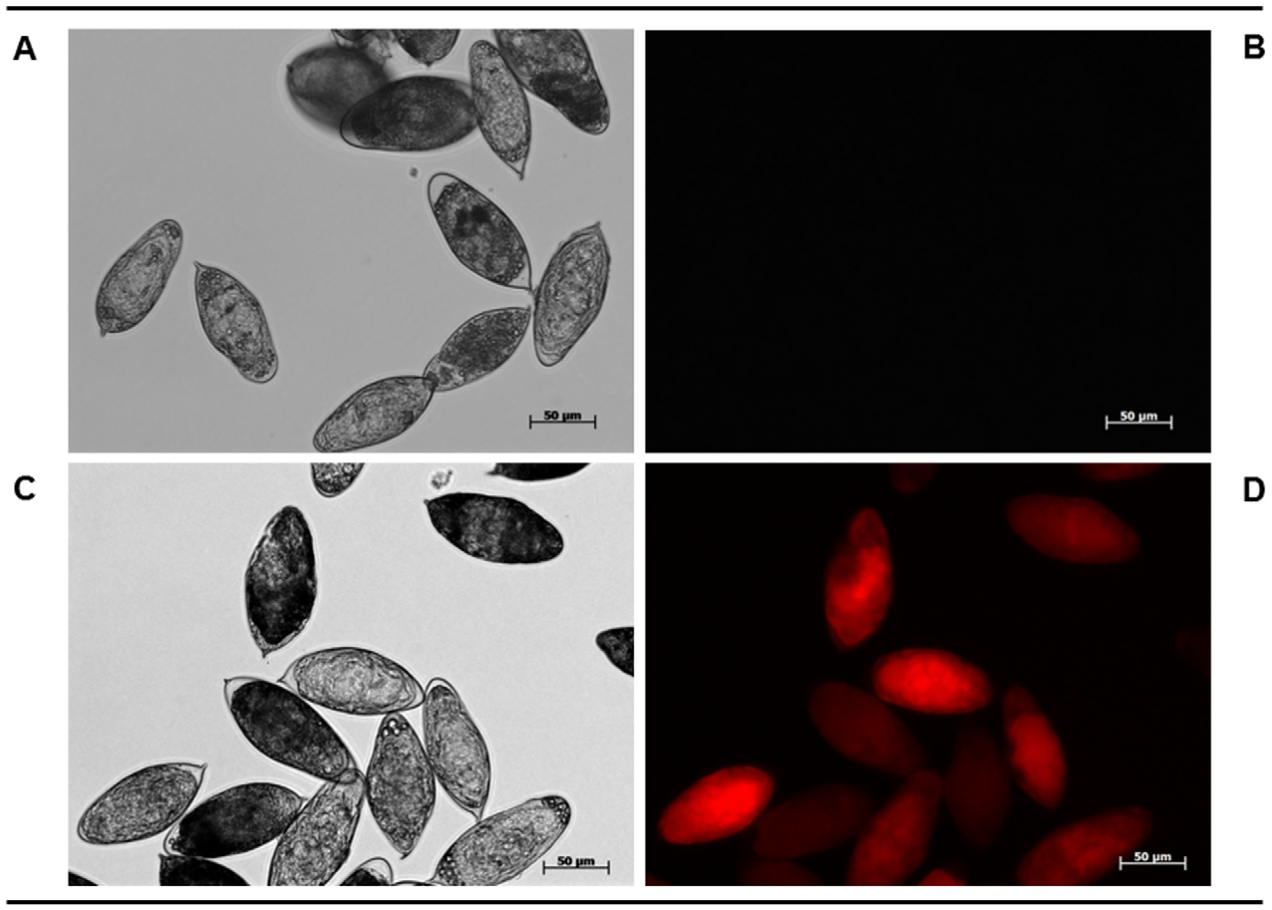
Labeled short interfering RNA enters cultured eggs of *Schistosoma haematobium*. Representative images of schistosome eggs 3 hours after soaking in Cy3-siRNA; panel A: no Cy3-siRNA treatment control, bright field; panel B: no Cy3-siRNA treatment control, fluorescence field, panel C: soaked eggs in medium containing 50 ng/µl of Cy3-siRNA, bright field, panel D: soaked eggs in medium containing 50 ng/µl of Cy3-siRNA, fluorescence field. Scale bar, 50 µm.

To investigate whether macromolecules could be introduced into *S. haematobium* eggs, cultures of eggs were incubated in a Cy3- siRNA (13.8 kDa) with or without concomitant square wave electroporation. Three hours after exposure to Cy3-siRNA, eggs were examined by fluorescence microscopy. Surprisingly, strong fluorescence including foci of intense fluorescence was revealed in Cy3-siRNA soaked eggs in contrast to those subjected to electroporation (Figure 3 and S1). More than 80% of the treated eggs emitted fluorescence as revealed at low magnification (Figure S1). These results indicated that it is possible to introduce Cy3-siRNA into *S. haematobium* eggs by simple soaking and that electroporation was not essential for this reporter probe. (However, with some stages, electroporation is more efficient: it mobilizes dsRNA or mRNA into the target worms quickly, which is advantageous when working with RNAs that are labile.) Although the structure of the *S. haematobium* eggshell is not well described, pores are present in the eggshell of *S. mansoni* eggs – the eggshell has been described as cribriform [28].

### Reporter firefly luciferase is active in *S. haematobium*

**Figure 4:**
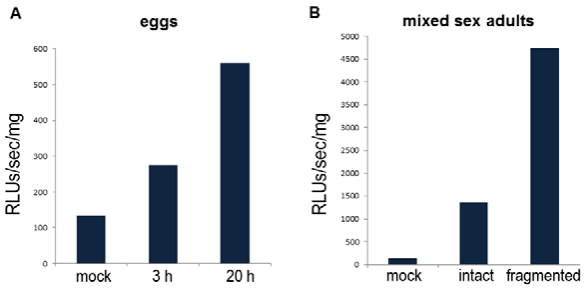
Luciferase activity measured in *Schistosoma haematobium.* Panel A: *S. haematobium* eggs transfected with 150 ng/µl of firefly luciferase mRNA. Detection of luciferase activity in mock control (mock) and in mLuc treated eggs, measured three (3 h) and 20 (20 h) hours after electroporation. Panel B: Luciferase activity measured in extracts of adult worms 3 h after electroporation, (mock) adult worms treated with no molecule, (intact) intact worms treated with 150 ng/µl mRNA, and (fragmented) worms diced into three or more pieces and treated with 150 ng/µl mRNA.

To ascertain if transgene mRNAs could penetrate schistosome eggs and be translated into an active protein, we electroporated cultured eggs in the presence of firefly luciferase mRNA (mLuc) (512 kDa). More specifically, 48 hours after isolation 1,500–2,500 eggs were subjected to electroporation in the presence of 150 ng/µl of mLuc, and collected three and 20 hours later. Luciferase activity was detected in the mLuc electroporated group compared with untreated control at 3 h, and even higher luciferase activity was measured in eggs harvested at 20 h after electroporation (Figure 4A). (A signal of ∼100–150 RLUs/sec/mg was measured in the mock control group, which represents the background baseline of this assay.) We electroporated intact and fragmented adult worms in the presence of 15 µg of luciferase mRNA, and measured the luciferase activity 3 hours later. Several fold (∼3.5 times) more activity was detected in fragmented than intact worms (Figure 4B), in like fashion to *S. mansoni* [24]. Collectively, these findings indicated that square wave electroporation efficiently delivered exogenous nucleic acids into the eggs and adults of *S. haematobium* and that reporter luciferase was functionally translated from this exogenous mRNA.

### dsRNA silences reporter luciferase mRNA in *S. haematobium*

**Figure 5:**
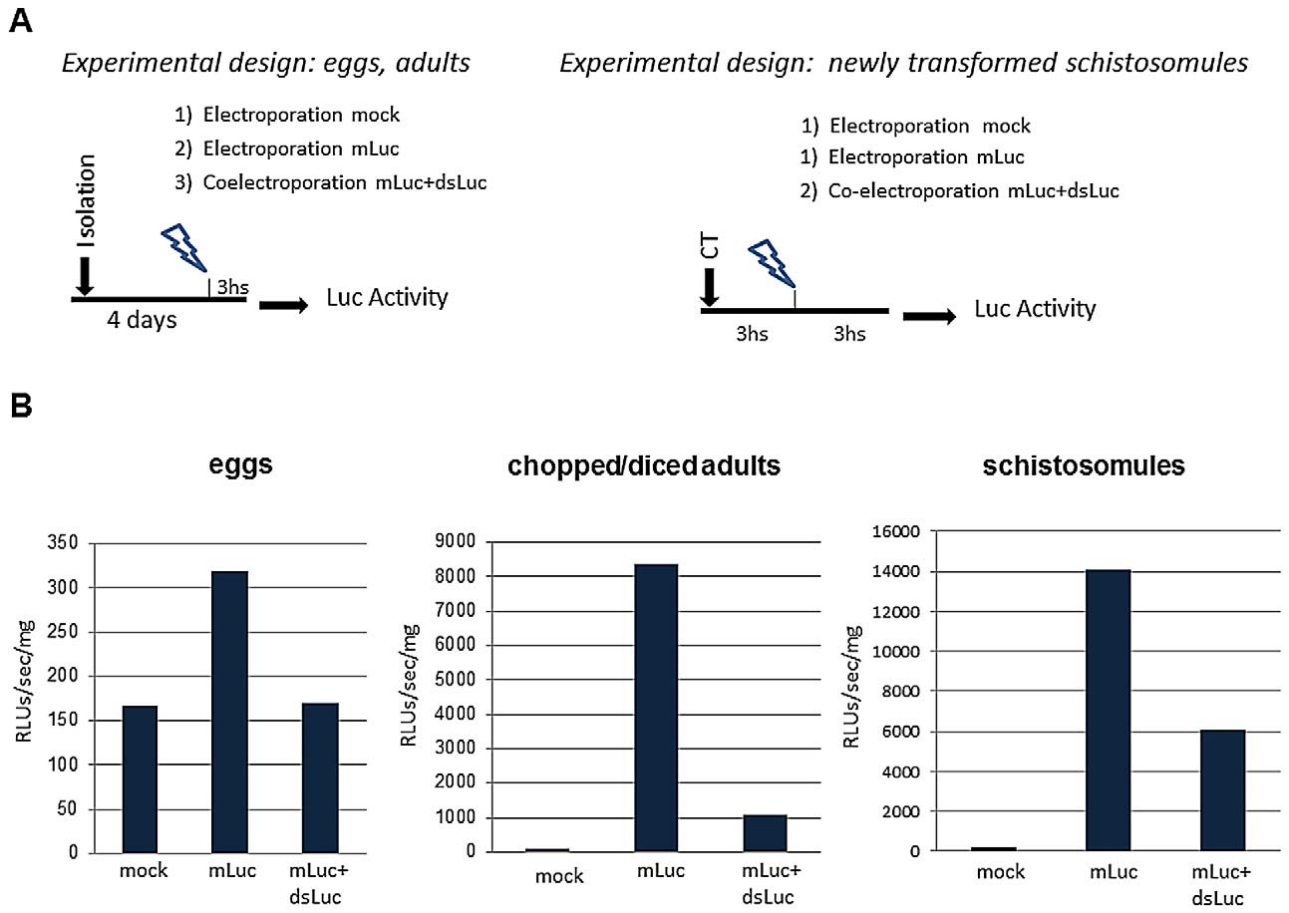
Suppression of exogenous luciferase activity in transfected eggs, chopped/diced adults and schistosomules of *Schistosoma haematobium*. Panel A: schematic representation of the experimental designs. Panel B: Luciferase activity measured in the indicated groups three hours after electroporation.

We have reported that it is feasible to knock down an exogenous reporter transgene by dsRNA in order to detect an active RNAi pathway in flukes [23], [29]. Given that *S. haematobium* can be productively transformed with mRNA by square wave electroporation, we proceeded to investigate silencing of expression of the exogenous reporter transcript (mLuc). About 2,000 eggs were removed from culture four days after isolation, washed and subjected to electroporation in the presence of both mLuc and dsLuc (mLuc+dsLuc group). Control eggs electroporated in the absence of exogenous RNAs (mock control) and positive control eggs electroporated in the presence of mLuc (mLuc group) were included (Figure 5A). Reduced luciferase activity was evident in the mLu*c*+dsLuc group in comparison with the mLuc group, even though the luciferase activity in terms of absolute RLUs/sec/mg measured in eggs at three hours after electroporation was relatively low in comparison to the other developmental stages (Figure 5B, left panel). (It appears to be more difficult to introduce mRNA into eggs than other developmental stages, likely because of the presence of the eggshell.) The experiment with eggs was repeated three times; knock down was apparent in two of the three trials. Fragmented adults were also examined; >75% knockdown of luciferase was observed (Figure 5B, center panel). The experiment was repeated; knock down was obvious on each occasion.

Furthermore, three hour old schistosomula were co-transfected with messenger RNA encoding luciferase (mLuc) and dsRNA targeting the luciferase transcript (dsLuc) by electroporation (mLu*c*+dsLuc group), along with controls (experimental design shown in Figure 5A). At 3 h after electroporation, luciferase activity of 14,080 RLU/sec/mg was evident in lysates of the positive control schistosomules transfected with mLuc. By contrast, luciferase activity in the schistosomules exposed to both dsLuc and mLuc was significantly lower, 6073 RLUs/sec/mg, representing 43% of the positive mLuc control (Figure 5B, right panel). In review, a similar trend was apparent in each of these developmental stages: it is feasible to knock down the reporter luciferase gene in eggs, schistosomules and adults of *S. haematobium.*

### Suppression of an endogenous gene in adults of *S. haematobium*

**Figure 6:**
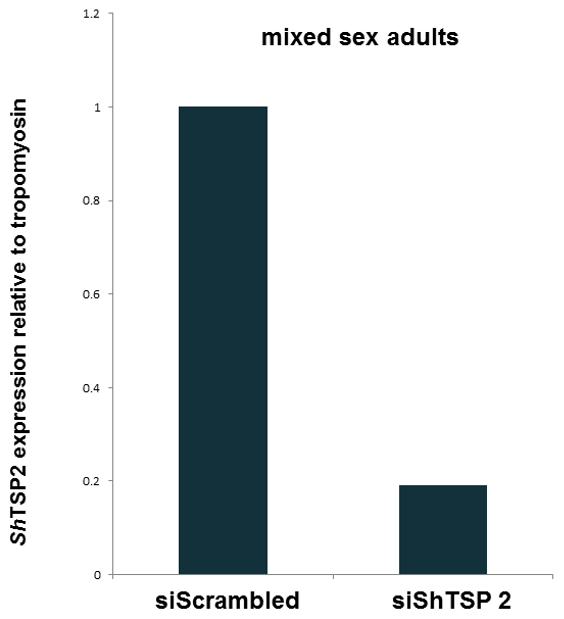
Silencing of the gene encoding the tetraspanin 2 antigen of *Schistosoma haematobium*. Quantitative RT-PCR analysis of the mRNAs from adult *S. haematobium* worms at 48 h after transfection by electroporation with siRNA specific for *Sh-tsp-2*. >80% silencing of the *Sh-tsp-2* (siShTSP2) evident when compared to the control group treated with siRNA scrambled control (siScrambled). *Sh-tsp-2* expression was normalized to a control mRNA encoding tropomyosin.

In addition to reporter luciferase, we introduced siRNA specific for *Sh-tsp-2*, an orthologue of a *S. mansoni* membrane protein critical for tegument formation, the tetraspanin *Sm*-TSP-2 [30] into intact *S. haematobium* mixed sex adults. At 48 hours after electroporation of siRNAs – si*Sh-tsp-2* and a control siRNA, we observed a significant knock-down of levels of the *Sh-tsp-2* transcript (Figure 6). This experiment was repeated three times; knock down was seen on each occasion, and on two of these three occasions the knock-down was >75%. Notably, no gross phenotypic differences among the adult worms were evident by light microscopy (not shown).

### Confocal micrographs highlight characteristic morphology of *S. haematobium*

**Figure 7:**
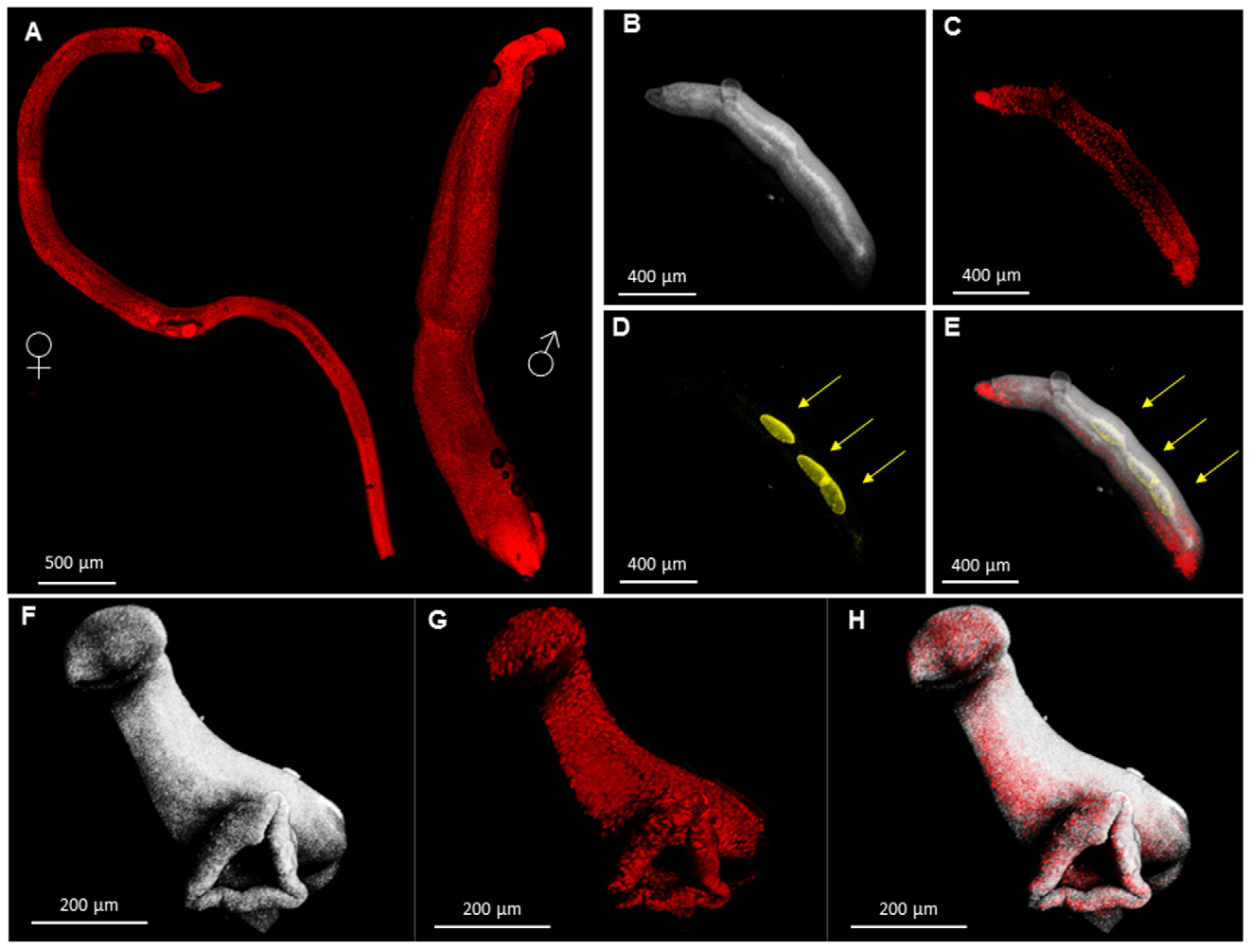
Representative three dimensional (3D) renderings from laser scanning confocal images of adult forms of *Schistosoma haematobium*. Panel **A**: 3D rendering from the female (left) and male (right) *S. haematobium*. Images were captured with 5× objective as tile-scan to cover the entire worm in addition to z-stacks. Propidium iodide (PI) was used to label the nuclei (red). **B–E**: High power (20×/1.0) 3D rendering from a female *S. haematobium* capturing the anterior of the worm. Three channels were extracted after applying a linear spectral unmixing algorithm to a lambda stack confocal images. **B**, The surface of the female *S. haematobium* visualized using reflected light scattering from the 488 nm laser line. **C**, PI –labeling, **D**, ‘autofluorescence’ from the schistosome eggs, **E**, merge of **B–D**, showing the structure of the anterior of the worm, with a semi-transparent visualization. In **D** and **E**, arrows indicate location of schistosome eggs. **F–H**, 3D rendering from the anterior of a male worm visualized with a similar approach used in **B–E**. **F**, reflected light channel, **G**, PI channel, **H**, merge of **F** and **G** using semi-transparent visualization. Scale bars, 500 µm (**A**), 400 µm (**B–E**), 200 µm (**F–H**).

In addition to the images of cultured stages of *S. haematobium*, adult worms were fixed in 4% paraformaldehyde and stained with PI. Spectral confocal microscopy was used to image the entire volume of the paraformaldehyde fixed male and female worms at high resolution (Figure 7A). We employed A T80/R20 beamsplitter to image the flukes, using backward scattered laser light. The reflected light is registered on the lambda stack as a dual-peak at the wavelength of the laser used for excitation. In this case, the 488 nm laser line produced a large reflection response, from which images of the surface of the schistosomes were assembled (Figure 7B and F). The approach also recorded in consistent and reproducible manner, autofluorescence deriving from the gut and, dramatically, eggs *in utero* (Figure 7D and E). The autofluorescence registered on the lambda stack displayed a broad spectrum - peak ∼560 nm, range 500–650 nm (overlapping with numerous widely employed dyes and fluorescent proteins). The signals from nuclei stained with PI (Figure 7C and G) registered as a spectral curve (peak 617 nm) that partially overlapped with the red-shifted slope of the autofluorescence. Thus, we could select discrete sites on worms representing reflected light, autofluorescence and PI fluorescence that served as references for linear unmixing [31]. The three-channel confocal stacks, derived after linear unmixing, comprised channels representing the reflection, autofluorescence and PI signal at high signal to noise ratio. Figures 7E and 7H show three-dimensional images assembled from the merged reflected light, autofluorescence and the PI fluorescence signals.

## Discussion

Using *S. haematobium* eggs from livers and intestines of experimentally infected hamsters, adult worms perfused from the hamsters and cercariae from *B. t. truncatus* snails, and using similar approaches to those for *S. mansoni* [20], we were able to culture eggs, schistosomules, and adults of *S. haematobium* and to subject these developmental stages to genetic manipulation. We transformed eggs of *S. haematobium* with a small nucleic acid probe, Cy3-siRNA. Experience with the other two major schistosomes has revealed that the schistosome egg represents an attractive developmental stage at which to target transgenes because it is readily obtained from experimentally-infected rodents or naturally infected people, is easily maintained in vitro, has a high ratio of germ to somatic cells and contains a miracidium that can be employed to infect snails to propagate the life cycle [21], [32], [33]. Furthermore, from the clinical perspective, the egg represents the major source of pathogenesis in human schistosomiasis haematobia. We observed that exogenous macromolecules penetrate into cultured eggs, and we speculate that small macromolecules such as Cy3-Silencer siRNA (13.8 kDa) enter eggs through the pores that likely anastomose throughout the eggshell and which provide access from sub-shell envelope and the developing miracidium to the exterior, in like fashion to the egg of *S. mansoni* [29], [34], [35].

Others and we have described the utility of firefly luciferase as a transgene probe in *S. mansoni* and the liver fluke *Fasciola hepatica* [21], [22], [23], [29], [36]. We have also reported the utility of luciferase as a model target to identify the presence of an active RNA interference pathway in less well studied helminth parasites, especially where genome sequences are unavailable [23]. Using this strategy, we now present findings that indicate for the first time the presence of an intact RNAi pathway in *S. haematobium*. In each of three developmental stages investigated – eggs, schistosomula, and mixed sex adults, co-introduction of dsRNA spanning the transcript of firefly luciferase and of mRNA encoding firefly luciferase resulted in robust knockdown of the exogenous mRNA. Luminometric measurement of luciferase activity provided a direct demonstration of gene silencing at the protein level.

In *S. mansoni*, comparative studies indicate that efficiency of RNAi efficiency following electroporation is superior to passive soaking [37]. Here we employed square wave electroporation to introduce the dsRNA and luciferase mRNA into developmental stages of *S. haematobium*. Eggs, schistosomula and adults of *S. haematobium* were amenable to transfection with foreign nucleic acids using this technique. Given that these stages tolerated the electro-transfection conditions well, we anticipate that this technique can be optimized for genetic analysis and genomic manipulation of *S. haematobium*. Whereas soaking performed better than electroporation alone for eggs of *S. haematobium*, it will be worthwhile to employ electroporation followed by soaking of the transfected eggs, a combination that is superior to soaking alone in eggs of *S. mansoni* [21].

Although little is known about the protein encoding genes of *S. haematobium*, we obtained the sequence of *Sh-tsp-2*, an apparent orthologue of *Sm-tsp-2* which encodes a lead vaccine antigen for schistosomiasis mansoni [30]. By targeting the sequence encoding the extracellular loop 2 domain of this protein with a 19 nt siRNA, we observed strong knockdown of the *Sh-tsp-2* transcript in adult worms. Thorough studies targeting this gene are warranted given its performance as a vaccine antigen for *S. mansoni* infection and because of the integral role that *Sm*-TSP-2 plays in development, maturation or stability of the tegument [30].

We deployed laser scanning confocal microscopy to view the adult stage of *S. haematobium*. In addition to facilitating views of the entire worms (≥1 cm in length), the approach circumvents barriers to reliable fluorescence imaging of schistosomes, including the notion of *autofluorescence* of schistosome eggs, e.g. [38]. The provenance of signals from the eggs (and gut) aside, the phenomenon deserves deeper exploration since it has the potential to chart predictable anatomical landmarks of the schistosome that can facilitate microanalysis of schistosome organs and tissues. Future characterization of native fluorescence signals of schistosomes can be expected to be of interest. Unlike fixed worms, evaluation of the living worms critically depends on minimizing invasive approaches. Also, fluorescence labels suitable for living cells generally cause some perturbation of normal functions. Thus, a library of spectrally distinctive signals – including the signal from eggs reported here - can be expected to facilitate microscopic imaging of viable schistosomes. Collectively, spectral confocal imaging provided the technological capacity to document eggs in utero of this neglected schistosome by extracting their emission of autofluorescence. Also, we imaged adult *S. haematobium* worms by staining tegumental nuclei with propidium iodide, which allowed the assembly of the three dimensional structure of the blood fluke. The spectral confocal microscopy approaches allowed differentiation of a fluorochrome from natural signals, e.g. PI versus autofluorescence, and portends its likely utility for monitoring reporter genes such as green fluorescent protein in transgenic schistosomes.

In conclusion, this is the first report of genetic manipulation of *S. haematobium*. The procedures described here are expected to find application in determining the importance of *S. haematobium* genes. Whereas few sequences are yet available, there is now increasing interest in sequencing the *S. haematobium* genome. Tools and procedures for genetic and genomic manipulation of *S. haematobium* will soon be needed to determine the importance of prospective new gene targets for development of novel interventions.

## Supporting Information

Figure S1 **Representative micrographs at low magnification (4×) of** ***Schistosoma haematobium*** **eggs at three hours after exposure to Cy3-siRNA.** Panels A and B: control without Cy3-siRNA, bright (A) and fluorescence (B) fields; panels C and D: soaked eggs in medium containing 50 ng/µl of Cy3-siRNA, bright (C) and fluorescence (D) fields. Panels E and F: control electroporated eggs without Cy3-siRNA, bright (E) and fluorescence (F) fields, panels G and H: eggs electroporated in the presence of 50 ng/µl of Cy3-siRNA, bright (G) and fluorescence (H) fields. Scale bar, 200 µm.
(TIF)
