# Supplementary figures and images for: Genetic Manipulation of Schistosoma haematobium, the Neglected Schistosome
Source: PLoS Negl Trop Dis. 2011 Oct 11;5(10):e1348. doi: 10.1371/journal.pntd.0001348 (PMC3191139; doi:10.1371/journal.pntd.0001348)

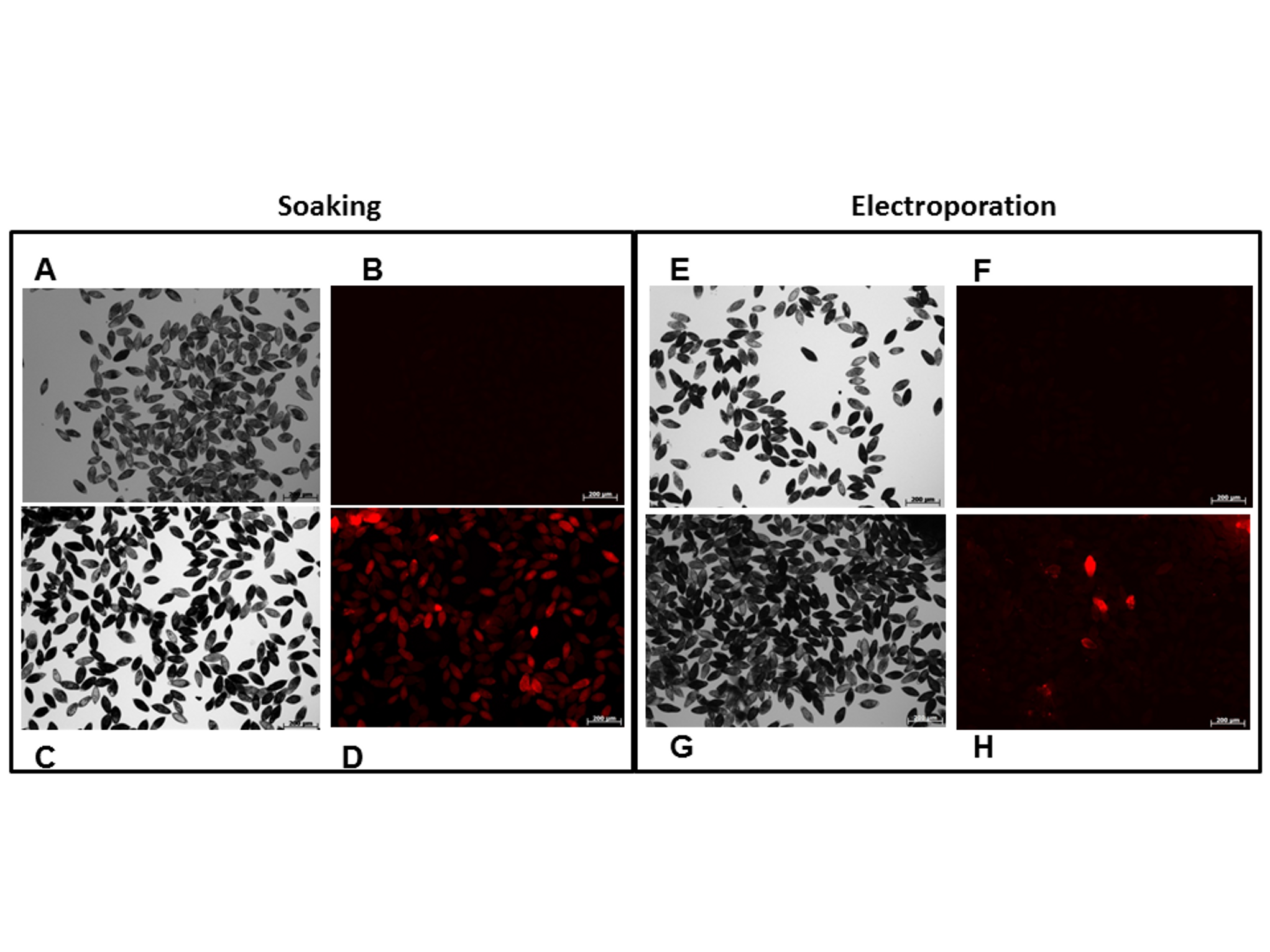

Supplement: Figure S1 — Representative micrographs at low magnification (4×) of Schistosoma haematobium eggs at three hours after exposure to Cy3-siRNA. Panels A and B: control without Cy3-siRNA, bright (A) and fluorescence (B) fields; panels C and D: soaked eggs in medium containing 50 ng/µl of Cy3-siRNA, bright (C) and fluorescence (D) fields. Panels E and F: control electroporated eggs without Cy3-siRNA, bright (E) and fluorescence (F) fields, panels G and H: eggs electroporated in the presence of 50 ng/µl of Cy3-siRNA, bright (G) and fluorescence (H) fields. Scale bar, 200 µm. (TIF) [file pntd.0001348.s001.tif]
